# Duplication cyst of the pylorus: a case report

**DOI:** 10.1186/1752-1947-7-175

**Published:** 2013-07-05

**Authors:** Kestutis Trainavicius, Pranas Gurskas, Jonas Povilavicius

**Affiliations:** 1 Children’s Surgery Centre, Children’s Hospital, Affiliate of Vilnius University Hospital Santariskiu Klinikos, Santariskiu Str. 7, LT 08406, Vilnius, Lithuania

**Keywords:** Gastrointestinal duplication, Gastrointestinal obstruction, Intra-abdominal organs cysts, Pyloric duplication cyst

## Abstract

**Introduction:**

Pyloric duplication is an extremely rare gastrointestinal tract malformation in neonates. This is the first case report of pyloric duplication in our country (Lithuania).

**Case presentation:**

We report the case of a 2-day-old Lithuanian girl who suffered from pyloric duplication mimicking an alternative common bile duct cyst or other intra-abdominal organs cysts. A laparotomy was performed and the cystic formation of the pyloric area was successfully resected. The postoperative course was uneventful.

**Conclusions:**

There are only a few reports describing abdominal masses caused by pyloric duplication mimicking common bile duct cyst or other intra-abdominal organs cysts. Therefore thorough clinical and instrumental examination is needed to determine the most accurate diagnosis that allows one to choose the right treatment.

## Introduction

The term “intestinal duplication” was first used by Fitz, but was not widely used until popularized by Ladd in 1937 [1]. Alimentary tract duplications are very rare and occur in approximately 1 out of 4500 births [2]. True pylorus duplications are extremely rare and present in approximately 2.2% of all alimentary tract duplications [3]. The female-to-male ratio is 2:1 [4,5]. Although digestive tract duplications are rare, small intestines are the most common location of malformations. Pyloric cystic duplication is the rarest alimentary tract duplication and the literature describes only a few cases. Duplication of the pylorus clinically manifests signs of gastric obstruction, making it difficult to distinguish from hypertrophic pyloric stenosis. The precise diagnosis of pyloric duplication is rarely made before surgery, because this alimentary tract malformation is extremely rare and difficult to perceive. The main method of treatment is radical resection.

## Case presentation

**Figure 1:**
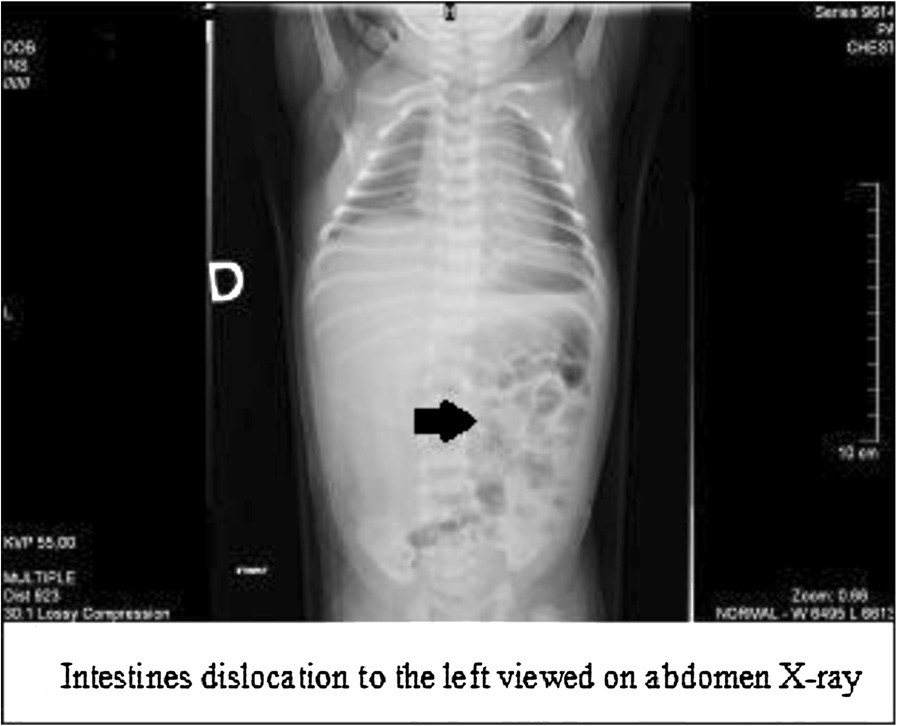
Dislocation of the intestines to the left (arrow) viewed on abdomen X-ray.

**Figure 2:**
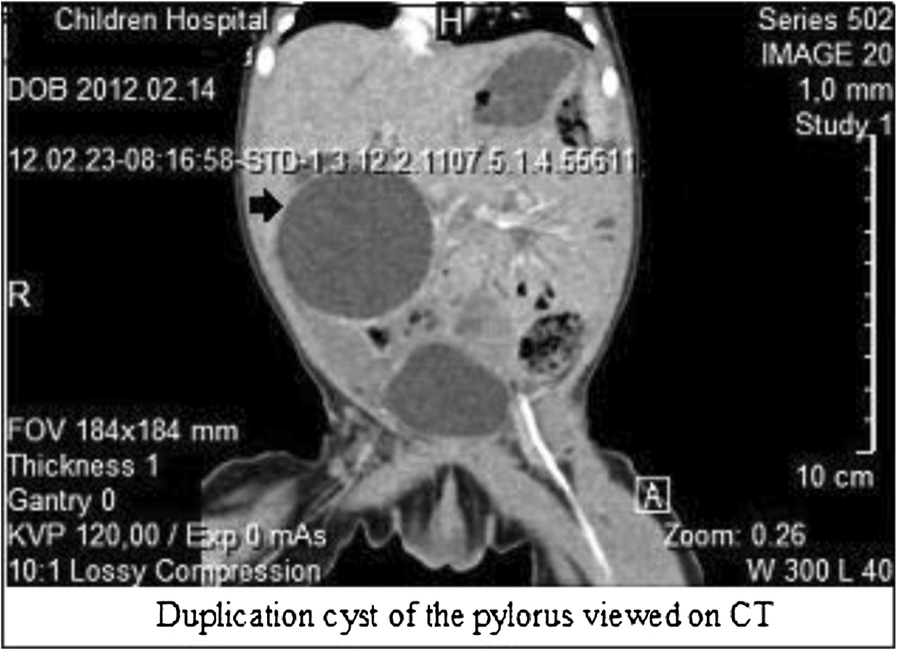
Duplication cyst of the pylorus (arrow) viewed on computed tomography (CT).

We present the case of a 2-day-old Lithuanian girl of third pregnancy and third birth who was born after a gestation period of 39 weeks. She was delivered by Cesarean section. Her weight at birth was 3450g and her Apgar score was evaluated as 10 out of 10 points. An ultrasound was done in the 23rd week of gestation and a cyst in the fetal abdominal cavity was detected. The newborn was admitted to our neonatal intensive care unit two days after birth because of vomiting. A palpable tumor was found in the upper right of her abdomen through inspection and palpation. An X-ray of her abdomen revealed dislocation of her intestines to the left side of the abdomen (Figure 1). Abdominal ultrasonography was performed after her birth. A 45mm diameter cystic tumor was found under her liver. A common bile duct cyst was suspected. An abdominal computed tomography (CT) revealed a cystic tumor under the liver measuring 48×49×44mm. A thin, smooth capsule of the cyst was silhouetted after contrasting. The tumor was pressing her gallbladder and was in close vicinity to her right kidney, dislocating her intestines to the left side of her abdomen (Figure 2). Repeated ultrasound indicated that the cyst had no connection to the biliary drainage channels.

**Figure 3:**
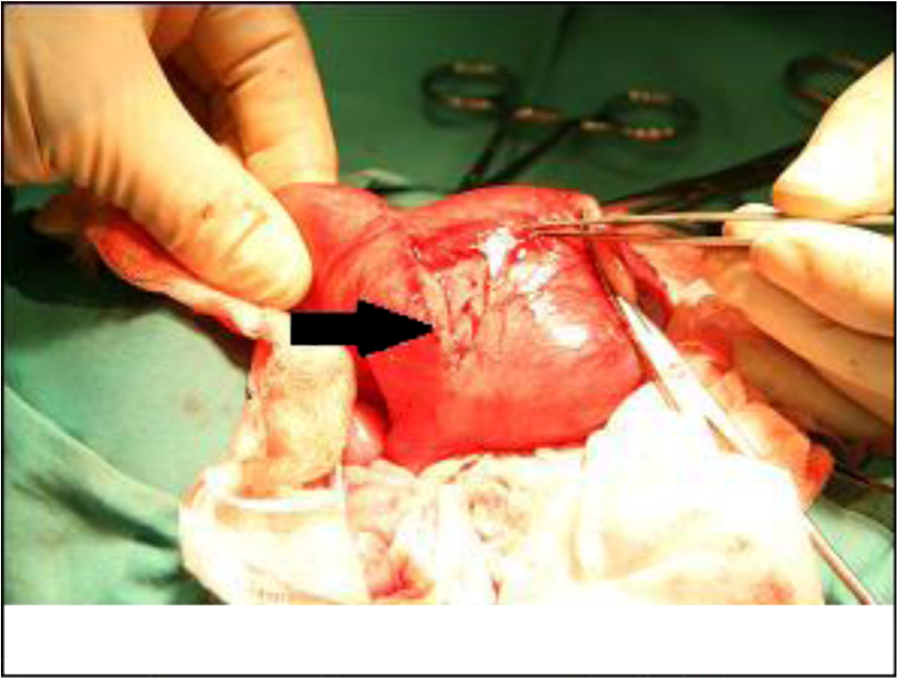
Duplication cyst of the pylorus (surgery picture).

The cyst began to grow and consequently symptoms of gastrointestinal obstruction began to present. Three weeks after her birth a laparotomy was performed. The cystic formation in the pyloric area was found and resected (Figure 3). Histopathological examination showed duplication cyst of the pylorus. The postoperative course was uneventful. The baby began to grow and gained weight after normal feeding resumed. The patient was discharged in good condition.

## Discussion

A duplication cyst of the pylorus is an extremely rare congenital anomaly of the gastrointestinal tract [6-9]. Duplications may occur in any part of the digestive tract [10], but they are most commonly found in the ileum, and rarely found in the pyloric area [2]. Pyloric duplications make up 2.2% of all alimentary tract duplications [3]. Of other gastrointestinal abnormalities and vertebral defects, 50% are accompanied by alimentary tract duplications [5]. This anomaly was first described by G.S. Ramsay in 1957 [11,12], and since then only a few cases of pyloric duplications have been described [6,12]. This is the first time this anomaly has been reported in Lithuania. The etiology of duplications is unclear. Bremer’s theory suggests that errors in recanalization occur during the fifth to sixth week of gestation, when epithelial proliferation obliterates the lumen of the intestines. Vacuoles form and coalesce to reestablish the lumen of the intestine. Duplications cause failure of these vacuoles to coalesce [13]. Of digestive tract duplications, 85% do not communicate with the gastrointestinal lumen [14]. Cysts near the pylorus, as in our case, are known to present in the neonatal period [4]. However, there are two gastrointestinal tract duplication cases reported in a 41-year-old and a 64-year-old patient [4]. Persistent vomiting is the main symptom of cysts near the pylorus, which occurs due to pyloric obturation [2,12,15-18]. Non-bilious vomiting simulating hypertrophic pyloric stenosis should be distinguished from a cyst near the pylorus [6-9]. The usual presentation of duplication of the pylorus is with abdominal mass, vomiting, weight loss and bleeding from the alimentary tract [4]. In older children, it may present with abdominal pain, gastrointestinal bleeding, fever, hemorrhage and perforation leading to peritonitis [19,20]. The diagnosis of pyloric duplication is difficult because of its rarity. Abdominal ultrasonography, contrast CT, nuclear magnetic resonance, X-ray and fibroesophagogastroduodenoscopy of the gastrointestinal tract facilitate the differential diagnosis, but the disease is most commonly diagnosed during surgery. The main method of treatment is the radical resection of the duplication without injury to the lumen of the bowel.

## Conclusions

1. Duplication cyst of the pylorus is an extremely rare congenital anomaly of the gastrointestinal tract.

2. Thorough clinical and instrumental examination helps to determine the most accurate diagnosis and allows one to choose the right treatment.

## Consent

A written informed consent was obtained from the parents of the patient for publication of this case report and accompanying images. A copy of the written consent is available for review by the Editor-in-Chief of this journal.

## Competing interests

The authors declare that they have no competing interests.

## Authors’ contributions

KT and PG performed the surgery, provided all operative details and photographic material. KT was a major contributor in editing, revising and final approval of the version of the manuscript to be published. PG contributed in patient care, acquisition of data and has been involved in drafting the manuscript. JP was a major contributor in writing and drafting of the manuscript, edited the final version of the manuscript, did the data analysis and reviewed the literature. KT, PG, and JP were involved in the conception and design, analysis of the data, preparation of the manuscript. All authors have given final approval of the version to be published.
